# Evaluation of Freezing-Induced Changes in Aroma Profiles of Pomegranate Juice by Quantitative Descriptive Sensory Analysis, Gas Chromatography–Mass Spectrometry/Olfactometry, Odor Activity Values, Orthogonal Partial Least Squares–Discriminant Analysis, and Odorant Addition Experiment

**DOI:** 10.3390/foods14101811

**Published:** 2025-05-20

**Authors:** Yixiu Chen, Peng Wang, Wanying He, Honglei Tian, Jingzhang Geng, Runguang Zhang, Ping Zhan

**Affiliations:** 1 College of Food Engineering and Nutritional Science, Shaanxi Normal University, Xi’an 710119, China; 2230038445@snnu.edu.cn (Y.C.); wangpeng@163.com (P.W.); hewanyinghzau@163.com (W.H.); sunshine@snnu.edu.cn (R.Z.); 2 College of Biological Science and Engineering, Shaanxi University of Technology, Hanzhong 723000, China; gengjingzhang@163.com

**Keywords:** pomegranate juice (PJ), freezing temperatures, sensory perception, aroma active compounds, freezing-induced changes

## Abstract

Freezing is employed to preserve the quality of pomegranate juice (PJ) for producing nutritious ready-to-drink beverages. However, the aroma compounds of PJ undergo significant alterations post-freezing. This study aimed to examine the impacts of different freezing temperatures (−20 °C, −40 °C, and −80 °C) on the flavor profiles of PJ, using fresh PJ as a control. The quantitative descriptive sensory analysis (QDA) results showed that grassy, woody, and fruity attributes of PJ were notably diminished following the freezing treatment. In total, 34 volatiles were identified by gas chromatography–mass spectrometry (GC-MS), of which 14 were perceived by GC–olfactometry (GC-O). Together with orthogonal partial least squares–discriminant analysis (OPLS-DA) and OAV tests, five volatiles were determined as key differential markers, and three (hexanol, (*Z*)-3-hexen-1-ol and *β*-myrcene) were selected for further odorant addition experiments. The results verified that (*Z*)-3-hexen-1-ol was the primary odorant for enhancing the grassy and fruity notes of PJ, while hexanol and *β*-myrcene were crucial for enhancing grassy and woody attributes, respectively.

## 1. Introduction

Pomegranate (*Punica granatum* L.) belongs to the *Punicaceae* family, and is a type of economic fruit [1]. China, one of the world’s largest producers, cultivates pomegranates mainly in Shaanxi, Henan, Shandong, and Sichuan Provinces, as well as in Chongqing Municipality [2]. Pomegranates are nutritionally valuable, containing abundant organic acids, carbohydrates, vitamins, polysaccharides, polyphenols, and essential minerals [3]. Studies have shown that pomegranate consumption is associated with a reduced risk of cardiac diseases, atherosclerosis, tumor formation, and cancer development [4]. However, its seasonal availability and perishability present significant storage and distribution challenges. As a result, the development of value-added, processed pomegranate products can help minimize waste and broaden market opportunities. One such product is pomegranate juice, which is typically extracted during the fruit’s harvest season and subsequently frozen to preserve its quality for the production of nutritious ready-to-drink beverages.

Freezing is a widely used method for extending the shelf life of juices. However, Kadri Koppel et al. [5] reported that freezing led to a decrease in the terpene content of pomegranate juice and reduced its sensory quality. Similarly, freezing temperatures have been shown to influence the aroma quality of frozen foods. For instance, Gou et al. [6] discovered that not-from-concentrate apple juice stored at −18 °C experienced more aroma loss than juice stored at −1.5 °C. Teng et al. [7] found that liquid nitrogen freezing at −80 °C preserved the flavor profile of Pacific oyster compared to other freezing groups (−20 °C, −40 °C, −60 °C, −100 °C, and −40 °C). The retention of volatile compounds in hand-grabbed mutton was demonstrated by -Luo et al. [8] to be more effective at −40 °C and −80 °C than at −18 °C, owing to faster freezing rates. To the best of our knowledge, the effect of freezing temperature on the aroma compounds of pomegranate juice has not been investigated.

Gas chromatography–mass spectrometry (GC-MS) is considered to be an effective technique for detecting volatiles in juice [9]. Gas chromatography–olfactometry (GC-O) integrates instrumental analysis with human olfaction to identify both the aroma characteristics and the intensity of aroma active compounds [10]. Recently, GC-MS combined with GC-O has been used to clarify the aroma of volatiles while detecting them. For example, Xiao et al. [11] utilized GC-MS and GC-O to analyze the volatiles of mandarin juice, showing that most ester compounds possess fruity and sweet aromas. Meanwhile, GC-MS and GC-O analyses determined that unsaturated aldehydes contribute to the grassy aroma in tomato juice [12]. Furthermore, mathematical analysis methods, such as odor activity values (OAVs), orthogonal partial least squares–discriminant analysis (OPLS-DA), and principal component analysis (PCA), can eliminate the subjectivity of sensory evaluation. The combination of GC-MS and OAV is particularly effective for screening the major aroma compounds in juices. Typically, volatiles with OAV > 1 are regarded as major contributors to the overall flavor. By applying GC-MS in conjunction with OAVs, Liu et al. [13] identified 14 major aroma compounds in watermelon juice. Sun et al. [14] employed GC-MS with PCA to differentiate not-from-concentrate and from-concentrate orange juices. Liu et al. [15] assessed the flavor compounds of fresh and heat-sterilized bayberry juice by GC-MS, identifying 18 differential volatiles through OPLS-DA. The above-mentioned studies demonstrate that GC-MS/O combined with OAV, PCA, and OPLS-DA can detect and discriminate volatile flavor profiles in pomegranate juices (either fresh or frozen).

The objectives of this study are as follows: (i) using GC-MS to investigate the variation in volatiles in fresh pomegranate juice under different freezing temperatures (−20, −40, and −80 °C); (ii) applying GC-O and OAVs to characterize the major aroma active compounds; and (iii) conducting OPLS-DA and odorant addition experiments to identify and validate the key differential aroma compounds. This study reveals the impacts of freezing on the flavor profile of pomegranate juice, and provides a theoretical basis for optimizing the freezing step in its processing. This study would offer valuable insights for the development of freezing technologies and the industrial production of pomegranate juice.

## 2. Materials and Methods

### 2.1. Chemicals

Authentic standards, used for volatile qualitative and quantitative analysis, were purchased from Sigma-Aldrich (Shanghai, China), including hexanal, (*E*)-2-hexenal, hexanol, nonanal, linalool, (*Z*)-3-hexen-1-ol, 1-octen-3-ol, heptanol, terpinen-4-ol, *α*-terpineol, *β*-pinene, *β*-myrcene, limonene, and *γ*-terpinene. All of these chemicals were of at least 97% purity. Additionally, 1,2-dichlorobenzene (≥99%, Sigma-Aldrich, (Shanghai, China)), applied as an internal standard (IS), and n-alkanes (C_7_–C_40_), employed to calculate the retention indices (RIs) of volatiles, were also supplied from Sigma-Aldrich (Shanghai, China).

### 2.2. Preparation of Pomegranate Juice (PJ) Samples

Fresh ‘Jinpitian’ pomegranates (60.0 kg) were collected from a local pomegranate orchard in Lintong District, Xi’an City, Shaanxi Province, China, in October 2023. Prior to juicing, all pomegranate fruits were thoroughly washed with distilled water and drained. The intact arils were manually separated from the whole fruit, subsequently subjected to manual pressing, and filtered through a quadruple-layered gauze to extract the pomegranate juice. The obtained juice was regarded as the control sample (fresh pomegranate juice, labeled as FPJ0), and immediately subjected to further analysis. Partial FPJ0 samples were immediately packed in glass bottles with the same weight (60.0 g), stored at −20 °C, −40 °C, and −80 °C, respectively, for 14 days, and then thawed at room temperature. The corresponding obtained samples were labeled FPJ_−20 °C, FPJ_−40 °C, and FPJ_−80 °C, respectively. After complete thawing, all three samples were immediately subjected to further analysis.

### 2.3. Quantitative Descriptive Sensory Analysis (QDA)

QDA was performed according to a previous study with minor modifications [16]. Eight well-trained flavor assessors (4 female and 4 male, 22–30 years) from the College of Food Engineering and Nutritional Science of Shaanxi Normal University participated in this study. All assessors were familiar with the evaluation procedure of QDA, and had professional abilities in evaluating the description and discrimination of juice aromas. Each pomegranate juice (20 mL) sample was placed in a 100 mL glass cup labeled with random triple digits, and evaluated by assessors in the sensory laboratory (at 25 °C, free of peculiar smells). Prior to sensory analysis, all assessors received 14 days of training to familiarize them with the aroma of the pomegranate juice. Subsequently, they discussed the aroma characteristics of pomegranate juice until they could agree on a common list of aromatic attributes and odor descriptors. Six odor descriptors, including fruity, grassy, woody, floral, earthy, and sweet, were finally adopted. The standard reference details for descriptor definition were presented in our previous study [17]. In addition, “favorability” was supplemented as a comprehensive metric for assessing the aroma quality of PJ samples. All samples were rated for odor intensities on a scale of 0–9 (0 = not perceivable, 1 = very weak, 3 = weak, 5 = moderate, 7 = strong, and 9 = extremely strong). Each sample was evaluated by each sensory assessor in triplicate. The average was taken as the final result.

### 2.4. Extraction of Volatiles by HS-SPME

Volatiles of pomegranate juice were extracted using HS-SPME according to the method of a previous report with modifications [18]. Pomegranate juice (8.0 g) was placed in a 20 mL headspace vial capped with a polytetrafluoroethylene–silicon septum. Then, 1.0 µL of internal standard (1,2-dichlorobenzene, 1.306 µg/µL) was added to the juice, resulting in a final concentration of 1.63 × 10^−4^ µg/kg in the juice. The vial with pomegranate juice was equilibrated at 36 °C in a thermostatic water bath for 15 min. The SPME fiber (75 µm CAR/PDMS/DVB extraction head, Bellefonte, PA, USA) was exposed to the vial headspace for 35 min, then immediately desorbed for 7 min in the GC inlet at 250 °C. Each sample was duplicated three times.

### 2.5. Identification of Volatiles Using GC-MS

The identification of volatiles was performed as reported previously with modifications [19]. The identification of volatiles was carried out by an Agilent GC-MS instrument (7890B-5977B, Agilent Technologies, Santa Clara, CA, USA) equipped with a DB-WAX capillary column (30 m × 0.25 mm × 0.25 μm, Agilent Technologies, USA). Helium (99.999%) was used as the carrier gas at a constant flow of 1.5 mL/min, and the GC inlet was set to splitless mode. The GC oven program was as follows: column temperature was initially set at 40 °C, held for 5 min, then ramped up to 120 °C at 5 °C/min for 10 min, before finally being increased to 220 °C at 5 °C/min and maintained for 5 min. The Mass Selective Detector conditions were as follows: The device was operated in electronic impact (EI) ionization mode with 70 eV electron impact energy. The mass range was 35–550 *m*/*z* with a scan frequency of 4.37 scans/s, and the ion source temperature was 230 °C.

### 2.6. GC-O Analysis

A sniffer (ODP 4; GERSTEL, Mülheim (Ruhr), Germany) coupled with GC-MS was employed to characterize the aroma active compounds. The effluent from the GC capillary column was split in a 1:1 ratio between the MS detector and the sniffing port. Moist air at 25 °C was circulated continuously at 40 mL/min during the olfactory process to prevent the assessors from drying out their nasal passages. The GC conditions were the same as above. Four evaluators (2 male and 2 female) were required to perceive the characteristics of the outflow gas and assess its aroma intensity (AI). To ensure the validity and accuracy of the GC-O results, each evaluator received professional training before the analysis by sniffing the solutions of reference compounds. The AI of each aroma compound was quantitatively measured on a scale ranging from 0 to 5 (where 0 = no aroma, 1 = weak, 3 = moderate, 5 = strong) [20].

### 2.7. Qualitative and Quantitative Analysis of Volatiles

Volatiles were first identified based on mass spectra matching with the standard NIST14 library, and then accuracy was proven by the retention indices (RIs) reported in the literature (NIST Chemistry WebBook, 2023). When available, volatiles were also identified by comparing the authentic standard compounds. The RI of each volatile was calculated from the retention time of n-alkanes (C_7_–C_40_) under identical experimental conditions. The external standard method was used to quantify the aroma active compounds of pomegranate juices. Each standard compound of known concentration spiked when the internal standard (1,2-dichlorobenzene, 1.306 µg) was mixed with the volatile-free pomegranate juice matrix (8.0 g). GC-MS was used to obtain the calibration curve. A similar volatile-free matrix was obtained by adding the same concentration of fructose as FPJ0 to Milli-Q deionized water, and adjusting its acidity to the same level as FPJ0 [19]. Standard calibration curves were established based on the ratio of the peak area of analyte to the internal standard (Y-axis) and the ratio of analyte concentration to the internal standard (X-axis). In addition, the concentrations of volatiles without an authentic standard were determined using an internal standard (1,2-dichlorobenzene).

### 2.8. Odor Activity Value (OAV)

OAVs were calculated by dividing the concentration of each aroma active compound in the samples by its detection odor threshold (OT) in water [21]. The OT values of different compounds in this study were acquired from the data reported in previous studies (listed in the footnote of Table 1. Generally, compounds with OAVs exceeding 1 are considered to be important contributors to sample flavor. In addition, a higher OAV of the compound signifies a greater contribution to the overall flavor.

### 2.9. Odorant Addition Experiment

Combining the OAV, VIP, and sensory results, the volatiles responsible for the sample aroma were screened out. On this basis, an odorant addition experiment was conducted on the key aroma active volatiles that disappeared in fresh FPJ0, aiming to further elucidate their contributions to PJ flavor. The detailed process is as follows: Initially, the above-selected volatiles were sequentially added to the frozen-treated samples (FPJ_−20 °C, FPJ_−40 °C, and FPJ_−80 °C), respectively, according to the natural concentration differences between FPJ0 and the 3 frozen samples. Then, trained panelists were tasked with evaluating these addition samples, which were then compared to the aroma profiles of the fresh control sample FPJ0 via the QDA method, as described in Section 2.3.

### 2.10. Statistical Analysis

The data presented in this study were collected via experiments that were performed separately in triplicate. Significant differences among samples were determined by analysis of variance (ANOVA) and Duncan’s multiple tests (*p* < 0.05) via IBM SPSS Statistics 26. Orthogonal partial least squares–discriminant analysis (OPLS-DA) was performed using SIMCA 14.1. The cluster heatmap was analyzed and plotted using TBtools v1.068. Origin 2022 was used to perform principal component analysis (PCA) and plot the other figures.

## 3. Results and Discussion

### 3.1. Aroma Profiles of Fresh and Frozen PJ

**Figure 1:**
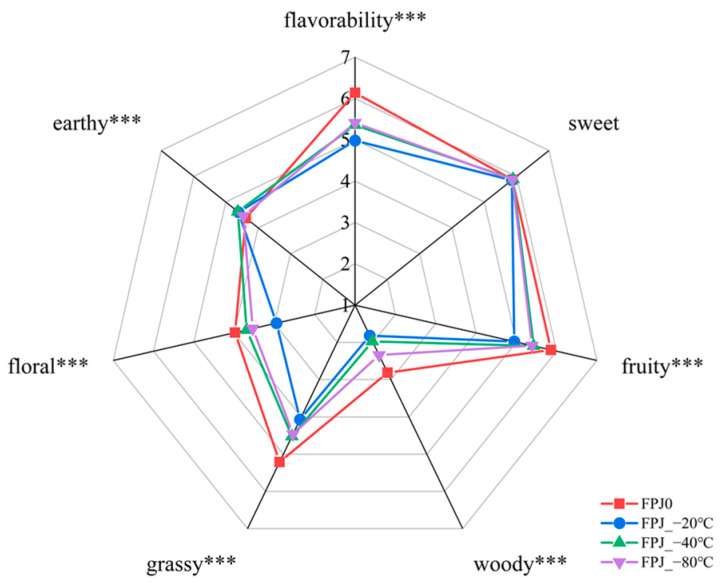
Aroma profiles of 1 fresh and 3 frozen-treated PJs, assessed by quantitative sensory descriptive analysis. “***”, extremely significant (*p* < 0.001).

QDA is a common approach for investigating the impacts of various processing treatments on the overall flavor profile of food products [17,23]. The aroma quality of four PJs, both before and after freezing, was assessed by QDA, as illustrated in Figure 1. Six attributes (fruity, sweet, woody, grassy, floral, and earthy) and one comprehensive matrix (favorability) were chosen for evaluating and comparing the different PJs. As shown, the fresh control sample FPJ0 showed the highest intensities of grassy, woody, fruity, and floral notes, but this was not found for the earthy aroma. In contrast, all frozen samples (−20, −40, and −80 °C) displayed an inverse trend: significant reductions in grassy, woody, fruity, and floral attributes (*p* < 0.001), accompanied by increased earthy notes (*p* < 0.001). This alteration may lead to a reduced favorability of frozen PJs. This phenomenon may be attributed to substantial volatile loss in PJs during frozen storage, leading to a progressive decline in grassy, woody, fruity, and floral aromatic intensities. The loss of volatiles can be caused by sudden extreme changes in temperature (both high and low), which can denature the chemistry, and thus change the aroma profile of the frozen sample [24]. However, no significant difference was observed in sweet notes between the fresh and frozen PJs, indicating that freezing minimally affected this characteristic note. Further, it was observed that the “favorability” attribute exhibited approximately three levels, with FPJ0 scoring the highest, followed by FPJ_−80 °C, FPJ_−40 °C, and FPJ_−20 °C, which received the lowest ratings.

### 3.2. Comparative Analysis of Volatiles Between Fresh and Frozen PJs

**Figure 2:**
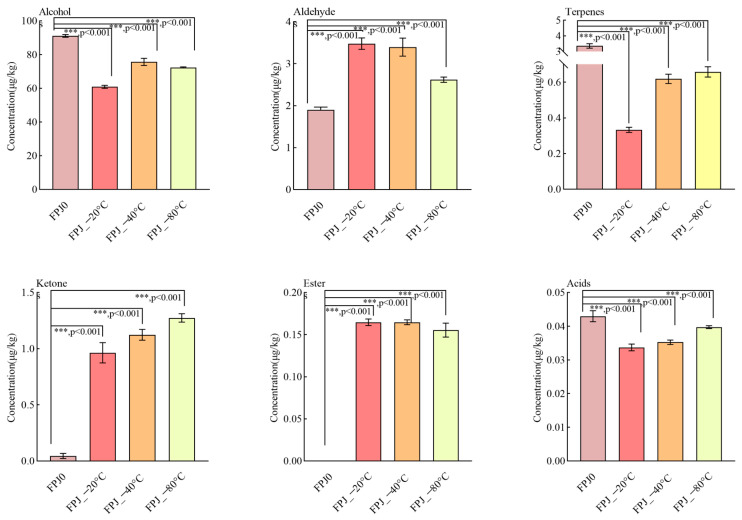
Concentrations of various compound types in FPJ and frozen pomegranate juice, measured by GC-MS. ***: significant at *p* < 0.001 level.

The volatiles of different frozen PJs (−20 °C, −40 °C, −80 °C) were analyzed and compared by HS-SPME-GC-MS, with the fresh juice sample FPJ0 as the control. A total of 34 volatiles were determined, including 7 terpenes, 7 aldehydes, 15 alcohols, 2 ketones, 2 esters, and 1 acid. Figure 2 shows the total contents of each group for the fresh and frozen PJs. As indicated, alcohols exhibited the highest contents (60.94–91.13 μg/kg), followed by aldehydes (1.90–3.48 μg/kg) and terpenes (0.28–3.32 μg/kg). This finding was similar to the previously reported data [25], which identified alcohols, aldehydes, and terpenes as the crucial volatiles in PJ. As shown in Figure 2, these characteristic volatiles of fresh PJs, mainly referring to alcohols and terpenes, were significantly reduced in the frozen samples. A similar declining trend was observed for acids. Conversely, the total contents of aldehydes, ketones, and esters increased remarkably after the freezing process. A detailed comparison of the major volatile groups considering the fresh and frozen PJs is presented below.

Alcohols, as the predominant contributors to PJ flavor, were derived from the oxidation of linoleic acid, and linolenic acid by lipoxygenase (LOX) [19,26]. As shown in Figure 2, alcohols suffered a decrease after the freezing process, ranging from 91.13 µg/kg in FPJ0 to 60.94 µg/kg, 75.66 µg/kg, and 72.39 µg/kg in the three frozen samples (−20, −40, and −80 °C, respectively). Among these, two C6 alcohols (hexanol and (*Z*)-3-hexen-1-ol) that imparted the fruity, floral, and grassy odor [27] were found in FPJ0 at relatively high levels of 67.76 ± 0.55 µg/kg and 20.45 ± 0.11 µg/kg, respectively (Table 2). However, these two compounds decreased significantly in the frozen samples and fluctuated remarkably as the frozen temperatures decreased. Linalool, reported as a pivotal contributor to the “floral” aroma of pomegranate [19], also exhibited a similar decrease in frozen samples (−20 °C, −40 °C, and −80 °C). Conversely, some terpene alcohols, such as terpinen-4-ol and *α*-terpineol, showed an increasing trend following the freezing process. This may be attributed to the degradation of terpenes into terpinen-4-ol and *α*-terpineol through acid-catalyzed and oxidation reactions [28,29]. Notably, *α*-terpineol seemed to be more easily formed at relatively low temperatures. As reported, this compound generally produced musty, stale, or piney notes [30]. The higher amounts of this compound in frozen samples possibly imparted an off-flavor in PJs.

In total, seven aldehydes were identified in the fresh and frozen PJ samples, and most of their levels in the frozen samples were remarkably higher than those in the fresh FPJ0. This increase in aldehyde content following freezing treatment aligned with the findings of Nukoon pupan et al. [24], who reported comparable aldehyde accumulation patterns in frozen durian puree. Hexanal, a C6 aldehyde responsible for “green” and “grass” odors [31], was found in FPJ0 at a relatively low concentration of 1.07 ± 0.06 µg/kg, while frozen-treated samples (FPJ_−20 °C, FPJ_−40 °C, and FPJ_−80 °C) exhibited higher contents, with the highest being in FPJ_−20 °C (3.03 ± 0.10 µg/kg), followed by 2.67 ± 0.12 and 2.10 ± 0.03 µg/kg in FPJ_−40 °C and FPJ_−80 °C, respectively. Octanal and nonanal levels in the frozen samples were also higher than in FPJ0. These two aldehydes were also described as having green, citrus, or fatty odors [32]. Increases in hexanal, octanal, and nonanal were expected to result in stronger grassy notes in the frozen samples (FPJ_−20 °C, FPJ_−40 °C, and FPJ_−80 °C). However, the sensory results (Figure 1) contradicted this prediction, with FPJ0 showing the highest grassy intensity. This discrepancy might be due to the presence of other grassy contributors in FPJ0, i.e., (*Z*)-3-hexen-1-ol. On the contrary, other aldehydes in fresh FPJ0 ((*E*)-2-hexenal, decanal, benzaldehyde, and undecanal) decreased significantly or became undetectable after freezing. Notably, the reduction pattern of (*E*)-2-hexenal mirrored that observed in pasteurized guava puree during frozen storage, suggesting a similar degradation behavior under frozen storage [33].

Terpene compounds were also identified as key factors in describing the fragrance of pomegranate fruit [34]. This group exhibited a similar decreased tendency to that of alcohols following the freezing treatment. Table 2 showed that seven terpenes were detected in the fresh control sample FPJ0, compared only with one, two, and four terpenes in FPJ_−20 °C, FPJ_−40 °C, and FPJ_−80 °C, respectively. The total terpene content in FPJ0 was 3.32 μg/kg, significantly higher than in frozen-treated samples. Similar results were found by Kadri Koppel et al. [5]. Limonene, the most abundant terpene in PJ, constituted up to 44.56% of the total terpene content in FPJ0. This compound was related to fruity characteristics (i.e., lemon, orange, and citrus odors) [35]. In contrast, the frozen-treated groups (−20, −40, and −80 °C) exhibited reductions in limonene content of 81.08%, 72.97.56%, and 71.62%, respectively, compared to FPJ0. In FPJ0, the other seven terpenes, including *β*-pinene, *β*-myrcene, *γ*-terpinene, *p*-cymene, trans-*α*-bergamotene, and *β*-copaene, exhibited either trace or even undetectable levels in the frozen groups. Most of these compounds were described as having woody and grassy flavor [11,36]. Consequently, the reduction or absence of these terpenes might result in weaker woody and grassy notes in frozen PJ samples.

With regard to esters, none were detected in FPJ0. Some certain esters, such as ethyl acetate and ethyl hexanoate, were identified as key contributors to the aroma of pomegranate [25,37]. However, these specific esters were not detected in FPJ0, possibly due to geographical and cultivar variations. Notably, following frozen storage, hexyl acetate was formed in all frozen samples (FPJ_−20 °C, FPJ_−40 °C, and FPJ_−80 °C), while (*Z*)-2-hexen-1-ol acetate was only present in FPJ_−80 °C, albeit at low concentrations. Similar to esters, ketones, including 6-Methyl-5-hepten-2-one and 2-nonanone, were also more abundant in the frozen samples. 6-Methyl-5-hepten-2-one, a significant contributor to herbaceous and green attributes of fruit [34], exhibited the highest content in FPJ_−40 °C, while 2-nonanone, described as having fresh and green notes [5,38], was only found in frozen samples, with FPJ_−80 °C having the highest amount (1.15 ± 0.03 µg/kg). In conclusion, the freezing treatment significantly altered the formation of volatile substances in pomegranate juice, with an overall decrease consistent with previous studies on the frozen storage of juice and fruit [6,39,40]. Compared to fresh sample FPJ0, the lowest fluctuation in volatiles was found in the FPJ_−40 °C and FPJ_−80 °C groups. This result was consistent with the QDA (Figure 1), which indicated that the FPJ_−40 °C and FPJ_−80 °C samples had less loss of aroma intensity, particularly for woody, grassy, fruity, and floral aromas.

**Figure 3:**
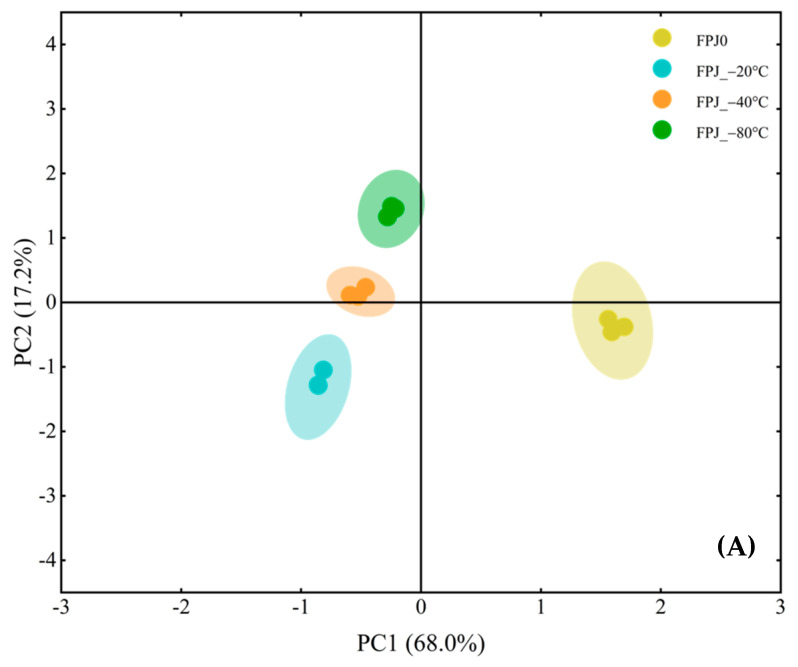

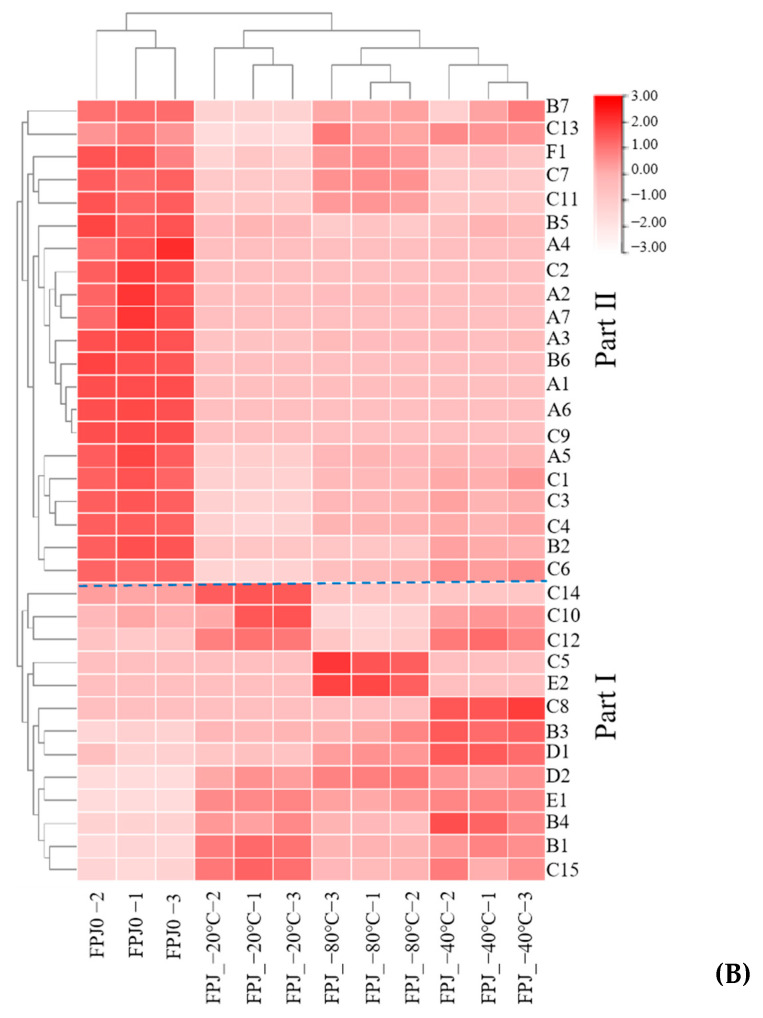
Multivariate statistical analysis of volatile compounds identified in fresh and frozen-treated PJ samples. (**A**) Score plot of principal component analysis (PCA); (**B**) hierarchical clustering heatmap (HCA) visualization (A1–F1 identified compounds as displayed in Table 2).

To explore the volatile differences between the fresh (FPJ0) and frozen PJ juices (FPJ_−20 °C, FPJ_−40 °C, and FPJ_−80 °C), principal component analysis (PCA) and hierarchical cluster analysis (HCA) based on all volatiles were further analyzed (Figure 3). Figure 3A shows the score plot of the PCA, with PC1 (68.0%) and PC2 (17.2%) contributing 85.2% of the total variance. As shown, along PC1, the fresh control sample FPJ0 and the three frozen PJ samples (FPJ_−20 °C, FPJ_−40 °C, and FPJ_−80 °C) are distinctly positioned on either side of the vertical axis. Among the frozen-treated samples, along PC2, three frozen samples are also clustered in different regions. These findings indicate that the volatile profiles of PJs post-freezing were significantly different from those of fresh juice. Figure 3B shows a heatmap of the hierarchical clustering analysis (HCA) based on Euclidean distance metrics. The relative concentrations of volatiles from low to high are represented through a normalized color gradation from a minimum of −3.00 (white) to a maximum of 3.00 (red). According to the dendrogram, the four PJ samples were clearly divided into two major clusters: FPJ0 was in one group, and FPJ_−20 °C, FPJ_−40 °C, and FPJ_−80 °C were in the other group, which was in accordance with the PCA results (Figure 3A), further indicating that the freezing process has a great impact on the volatile profiles of PJs. In addition, as for volatiles, they could also clearly be divided into two parts (I and II). The volatiles in Part I were relatively more abundant in the frozen samples, with a total of 13 volatiles, of which aldehydes and alcohols accounted for a larger proportion. Meanwhile, the relative contents of 21 volatiles in Part II were the highest in FPJ0, followed by FPJ_−80 °C and FPJ_−40 °C, and finally FPJ_−20 °C. Among them, terpenes and alcohols accounted for the main proportion. Overall, the composition of volatiles varied greatly in the fresh and frozen samples.

### 3.3. Analysis of Aroma Active Compounds in Fresh and Frozen PJs Through GC-O/AI and OAV

Numerous studies indicate that a multitude of volatiles have been identified within the food matrix, yet only a few contribute to aroma perception [41,42]. It is noteworthy that certain volatiles, even at low concentrations, should be paid more attention due to their extremely low odor thresholds. For this, the aroma active compounds present in the PJs were identified and screened out using GC–O/AI and odor activity value (OAV) analyses.

**Figure 4:**
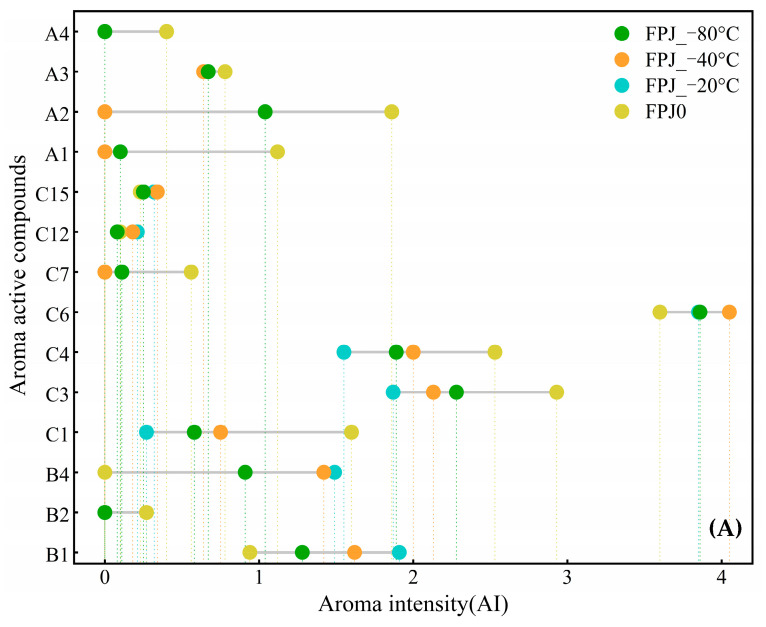

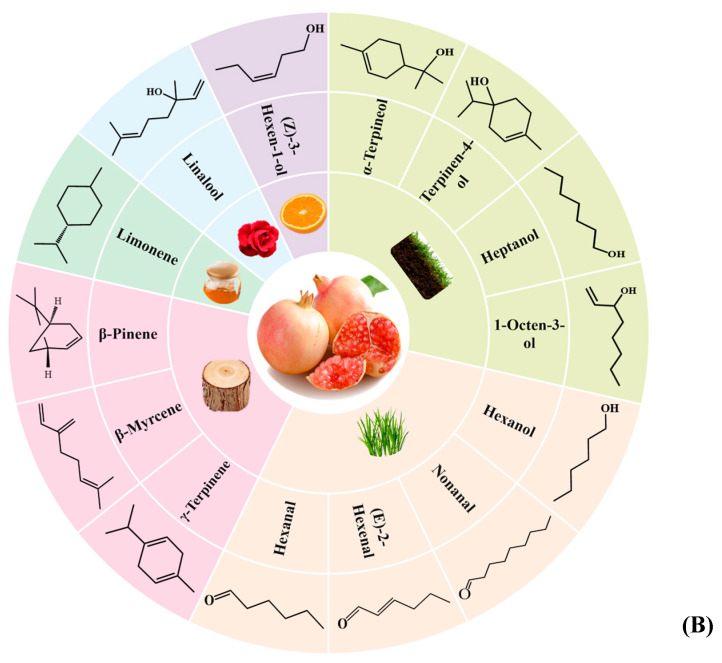
Aroma active compounds identified in FPJ0 and 3 frozen samples (A1–C15 identified compounds as displayed in Table 2). (**A**) Aroma intensity of aroma active compounds. (**B**) Odor attributes of aroma active compounds.

As is known, odor-specific magnitude estimation (OSME) is widely employed to assess the volatiles responsible for aroma perception based on their aroma intensity (AI). Typically, higher AIs are attributed to more intense aromas, and are thus regarded as more significant to the aroma profiles of food. The application of GC-O/AI to different PJs showed a total of 14 aroma active compounds. Seven alcohols, four terpenes, and three aldehydes were perceived. The detailed data are indicated in Figure 4. These perceived compounds were all identified based on MS, RIs, and standards. As shown, the control group (FPJ0) contained 13 aroma active compounds, whereas the FPJ_−20 °C, FPJ_−40 °C, and FPJ_−80 °C samples contained only 9, 9, and 12 compounds, respectively. According to their characteristics, these compounds can be classified into five categories. Class 1 ((*Z*)-3-hexen-1-ol) was characterized by fruity notes; Class 2, comprising hexanal, (*E*)-2-hexenal, nonanal, and hexanol, was primarily associated with a grassy odor; Class 3, consisting of heptanol, terpinen-4-ol, *α*-terpineol, and 1-octen-3-ol, was perceived as having earthy notes; Class 4, including *β*-pinene, *γ*-terpinene, and *β*-myrcene, was described as having woody notes; and Class 5 (linalool, limonene) featured floral or sweet descriptors. These findings aligned with previous research by Lu et al. [19], who similarly identified *β*-pinene and *β*-myrcene as woody aroma components in pomegranate juice. Among these, nine odorants ((*E*)-2-hexenal, linalool, hexanol, (*Z*)-3-hexen-1-ol, heptanol, *β*-pinene, *β*-myrcene, *γ*-terpinene, and limonene) exhibited the near-highest AIs in the fresh control sample FPJ0, potentially representing the primary contributors to PJ flavor. However, the AIs of most compounds exhibited decreasing trends following frozen storage. By contrast, four compounds, 1-octen-3-ol, *α*-terpineol, terpinen-4-ol, and nonanal, were found to have relatively strong intensities in the frozen-treated samples. The differential AIs of these 14 aroma active compounds among the PJs would result in significantly different sensory profiles.

Unlike GC–O analysis, OAV can take the influence of the food matrix into account, as the odor thresholds involved depend on the individual food matrix [43]. Thus, OAVs are extensively employed in aroma active compound recognition [35,44]. To delve deeper into their contribution to PJ aroma, the compounds perceived via GC-O/AI were selected for OAV determination. Commonly, OAVs are calculated based on their natural concentrations, with the aid of standard curves and odor thresholds sourced from the literature [22]. As shown, of the 14 aroma active compounds identified by GC-O/AI, the OAVs of six, six, six, and seven volatiles were more than 1 in FPJ0, FPJ_−20 °C, FPJ_−40 °C, and FPJ_−80 °C, respectively.

Among them, 1-octen-3-ol, 1-hexanol, (*Z*)-3-hexen-1-ol, hexanal, nonanal, and *β*-myrcene were confirmed as potent contributors to pomegranate juice’s aroma profile in previous studies [19,36]. Seven volatiles perceived by GC-O were not identified through OAV analysis, possibly attributed to their high odor thresholds. Among the potential odorants, as the crucial characteristic contributors to PJs, hexanol and 1-octen-3-ol accounted for the highest OAVs in all samples, ranging from 158.725 to 231.231 and from 375.550 to 450.044, respectively. Similar results were found by Lu et al. [19]. The OAVs of hexanal and (*Z*)-3-hexen-1-ol, which are responsible for grassy and fruity notes, also had OAVs greater than 1. Of note, linalool, endowing a floral aroma, was dominant in the fresh control sample FPJ0, which is consistent with previous reports [19]. However, the OAVs of this volatile were decreased in frozen PJs (FPJ_−20 °C, FPJ_−40 °C, FPJ_−80 °C). This was consistent with the GC-O/AI analysis, and may explain the attenuated floral intensity in frozen samples. Similarly, *β*-myrcene was also undetected or sharply decreased during the freezing process.

### 3.4. Key Differential Aroma Active Compounds Between FPJ0 and Frozen Samples

**Figure 5:**
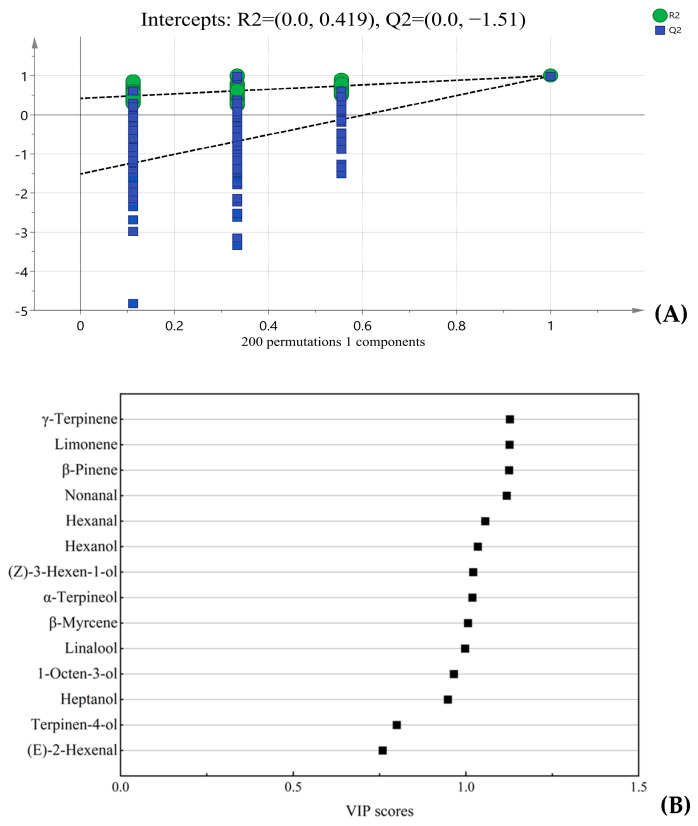
OPLS-DA analysis of FPJ and frozen juices. (**A**) Cross-validation of the OPLS-DA model. (**B**) VIP scores obtained from OPLS-DA analysis of FPJ and frozen juices.

To further explore the key aroma active compounds differentiating FPJ0 and frozen juices, a supervised OPLS-DA was performed. The compounds perceived via GC-O/AI were selected for OPLS-DA. In OPLS-DA model, the reliability and prediction ability of the model were assessed by *R2* (y) = 0.998 and *Q2* = 0.991. Another permutation test (*n* = 200) checked the reliability of the model (Figure 5A). Both the indexes indicated that the OPLS-DA model had good reliability without over-fitting. Subsequently, the variable importance in projection (VIP) values of the aroma active compounds were calculated (Figure 5B). Typically, variables with VIP > 1 play important roles in distinguishing various samples. As shown, the key markers distinguishing FPJ0 from the frozen samples included *β*-myrcene, (*Z*)-3-hexen-1-ol, hexanol, α-terpineol, hexanal, nonanal, *β*-pinene, limonene, and *γ*-terpinene.

Based on OAV > 1 and VIP > 1, five compounds (nonanal, hexanal, hexanol, (*Z*)-3-hexen-1-ol, and *β*-myrcene (Figure 5B)) were ultimately considered as the key differential volatiles between FPJ0 and frozen samples. Among these, the last three compounds (hexanol, (*Z*)-3-hexen-1-ol, and *β*-myrcene) were the ones that significantly decreased or even disappeared in the three frozen samples. Consequently, they were selected as the key differential aroma active compounds for further addition experiments.

### 3.5. Odorant Addition Experiment

**Figure 6:**
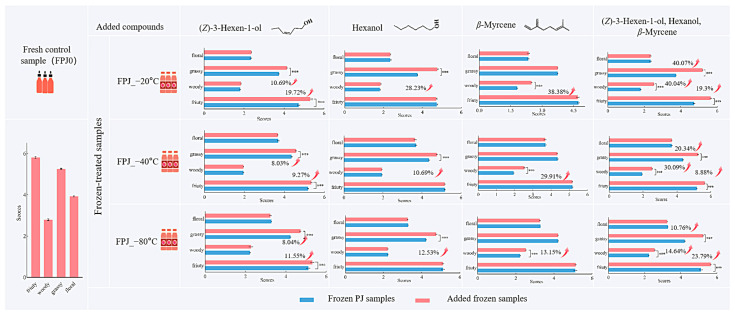
The aroma intensity achieved by adding hexanol, (*Z*)-3-hexen-1-ol, *β*-myrcene, and their mixtures to the three frozen-treated juices. ***: significant at *p* < 0.001 level, arrow: the aroma intensity increased significantly with the addition of the substance.

A series of controlled odorant addition experiments was conducted to further validate the contribution of key differential aroma active compounds. As indicated by the QDA results (Figure 1), the primary flavor differences between FPJ0 and the frozen-treated samples were characterized by grassy, fruity, floral, and woody attributes. Consequently, these four sensory attributes were selected for evaluating the aroma profiles of the samples, with the fresh sample FPJ0 as the control. The three frozen-treated samples were utilized as carriers for odorant addition experiments. Compound selection was based on two essential criteria: (1) OAV and VIP scores exceeding 1.0, and (2) concentration changes after freezing that correlated with the observed alterations in the aroma attributes mentioned above. While nonanal and hexanal satisfied the first criterion (OAV/VIP > 1), their concentration profiles showed a significant increase post-freezing, which contrasted with the depletion pattern of our target compounds and the diminishing trends of the associated sensory attributes. Consequently, these compounds were excluded from subsequent analyses. Finally, three individual key aroma active compounds (hexanol, (*Z*)-3-hexen-1-ol and *β*-myrcene) and their mixtures were sequentially added to the frozen samples (FPJ_−20 °C, FPJ_−40 °C, and FPJ_−80 °C) at concentrations corresponding to their natural differences from FPJ0. The results are shown in Figure 6. The addition of these volatiles remarkably enhanced one or more sensory attributes of the frozen-treated samples. For example, the addition of (*Z*)-3-hexen-1-ol resulted in a significant enhancement of grassy and fruity notes (*p* < 0.001). Specifically, the grassy scores in three frozen-treated samples (FPJ_−20 °C, FPJ_−40 °C, and FPJ_−80 °C) increased by 10.69%, 8.03%, and 8.04%, respectively. The intensity of the fruity attribute raised by 19.72%, 9.27%, and 11.55% after adding (*Z*)-3-hexen-1-ol, respectively. Additionally, the incorporation of hexanol into the frozen-treated juices also significantly increased the grassy attribute (*p* < 0.001), with increases of 28.23%, 10.69%, and 12.53%, with the corresponding frozen-treated samples (FPJ_−20 °C, FPJ_−40 °C, and FPJ_−80 °C) as controls, respectively. *β*-Myrcene, a volatile of interest for its woody characteristics, also resulted in significant increases in woody scores by 38.38%, 29.91%, and 13.15% in the three frozen-treated PJ samples, respectively. Further, the frozen-treated PJ samples with the three key aroma volatiles added (hexanol, (*Z*)-3-hexen-1-ol, and *β*-myrcene) resulted in scores of 5.24 (FPJ_−20 °C), 5.25 (FPJ_−40 °C), and 5.24 (FPJ_−80 °C) for the grassy note; 2.53 (FPJ_−20 °C), 2.54 (FPJ_−40 °C), and 2.56 (FPJ_−80 °C) for the woody note; and 5.65 (FPJ_−20 °C), 5.64 (FPJ_−40 °C), and 5.66 (FPJ_−80 °C) for the fruity note. These scores represented increases of 40.07%, 20.34%, and 10.76% for the grassy note; 40.04%, 30.09%, and 14.64% for the woody note; and 19.3%, 8.88%, and 23.79% for the fruity note in the corresponding added groups. Compared to the fresh sample FPJ0, the added frozen PJs exhibited minimal differences in grassy, woody, and fruity notes. These findings indicated that (*Z*)-3-hexen-1-ol, *β*-myrcene, and hexanol could partly restore the grassy, fruity, and woody attributes of the frozen samples. Thus, these compounds were considered as the primary causes of the differences in aroma profiles between the fresh and frozen-treated PJ samples.

## 4. Conclusions

Fresh and frozen-treated PJ samples were compared and analyzed for their aroma profiles and volatile compositions. The QDA results found significant differences in sensory attributes between the fresh and frozen PJs, especially for the grassy, woody, and fruity notes. A total of 34 odorants were screened out in four PJs by GC-MS-O. Among which, FPJ_−40 °C and FPJ_−80 °C appeared to retain more aroma compounds than FPJ_−20 °C. Based on VIP (>1) and OAVs (>1), five odorants—nonanal, hexanal, hexanol, (*Z*)-3-hexen-1-ol, and *β*-myrcene—were finally identified as key differential markers distinguishing fresh from frozen PJs, and the last three odorants were selected to conduct further odorant addition experiments. The results confirmed that the increased concentrations of hexanol, (*Z*)-3-hexen-1-ol, and *β*-myrcene were primarily responsible for the enhanced grassy, fruity, and woody notes in the frozen PJ samples. This study provides valuable insights into the sensory quality and aroma profiles of frozen PJs. In the future, the impacts of other factors, such as freezing time, thawing temperature, and processing conditions, on aroma characteristics of PJs will be further examined in order to develop a scientific and reasonable freezing method that effectively preserves the natural flavor of fresh PJ.

## Figures and Tables

**Table 1:** Calculation of concentrations and odor activity values (OAVs) of important aroma active compounds in fresh pomegranate juice (FPJ) and frozen juices.

Aroma Active Compounds	Linear Equations	R^2^	OTs (µg/kg) ^a^	Concentration (μg/kg)				OAVs			
				FPJ0	FPJ_−20 °C	FPJ_−40 °C	FPJ_−80 °C	FPJ0	FPJ_−20 °C	FPJ_−40 °C	FPJ_−80 °C
Hexanal	y = 0.0667x − 0.1147	0.9963	6.9	19.215	48.196	42.792	34.348	2.785	6.985	6.202	4.978
(*E*)-2-Hexenal	y = 0.0869x − 0.0279	0.9999	17	1.121	0.000	0.815	0.000	0.066	0.000	0.048	0.000
Nonanal	y = 0.0144x + 0.0123	0.9998	1	0.000	5.041	5.559	4.493	0.000	5.041	5.559	4.493
Linalool	y = 0.0899x − 0.1	0.9984	0.22	6.029	1.917	4.308	3.542	27.403	8.714	19.583	16.101
Hexanol	y = 0.0524x − 0.0539	0.9967	5.6	1294.896	888.859	1089.149	1044.915	231.231	158.725	194.491	186.592
(*Z*)-3-Hexen-1-ol	y = 0.1119x − 0.1106	0.9988	3.9	183.150	101.996	143.198	137.825	46.962	26.153	36.717	35.340
1-Octen-3-ol	y = 0.0993x − 0.1079	0.997	0.005	1.878	2.013	2.250	2.068	375.550	402.629	450.044	413.520
Heptanol	y = 0.0816x − 0.0391	0.9996	5.4	2.211	0.000	0.000	1.100	0.409	0.000	0.000	0.204
Terpinen-4-ol	y = 0.1047x − 0.0764	0.9985	340	1.677	2.566	2.558	1.540	0.005	0.008	0.008	0.005
*α*-Terpineol	y = 0.1355x − 0.1672	0.9967	280	11.714	16.262	15.098	13.792	0.23	0.32	0.34	0.25
*β*-Pinene	y = 0.0965x − 0.0598	0.9993	140	10.684	0.000	0.000	1.285	0.076	0.000	0.000	0.009
*β*-Myrcene	y = 0.0761x − 0.1092	0.9972	1.2	4.962	0.000	0.000	2.494	4.135	0.000	0.000	2.078
Limonene	y = 0.0103x − 0.0598	0.9996	10	1.844	1.109	1.181	1.192	0.184	0.111	0.118	0.119
*γ*-Terpinene	y = 0.1022x − 0.0758	0.9999	65	2.256	0.000	0.000	0.000	0.035	0.000	0.000	0.000

^a^ OT: The odor threshold values of flavor compounds were obtained from the book [22].

**Table 2:** The content of compounds measured by GC-MS in fresh and frozen PJs.

No.	Compounds	Identification	RIa	RIb	FPJ0	Frozen-Treated Samples		
						FPJ_−20 °C	FPJ_−40 °C	FPJ_−80 °C
A1	*β*-Pinene	RI MS St	1096	1110	0.93 ± 0.01 a	ND	ND	0.03 ± 0.00 b
A2	*β*-Myrcene	RI MS St	1148	1146	0.20 ± 0.03 a	ND	ND	0.01 ± 0.00 b
A3	Limonene	RI MS St	1183	1160	1.48 ± 0.03 a	0.28 ± 0.01 c	0.40 ± 0.02 b	0.42 ± 0.02 b
A4	*γ*-Terpinene	RI MS St	1216	1205	0.11 ± 0.02	ND	ND	ND
A5	*p*-Cymene	RI MS St	1244	1259	0.43 ± 0.03 a	ND	0.14 ± 0.00 b	0.14 ± 0.01 b
A6	trans-*α*-Bergamotene	RI MS	1575	1573	0.06 ± 0.00	ND	ND	ND
A7	*β*-copaene	MS	1588	1603	0.08 ± 0.01	ND	ND	ND
B1	Hexanal	RI MS St	1080	1102	1.07 ± 0.06 d	3.03 ± 0.10 a	2.67 ± 0.12 b	2.10 ± 0.03 c
B2	(*E*)-2-Hexenal	RI MS St	1200	1201	0.06 ± 0.00 a	ND	0.03 ± 0.00 b	ND
B3	Octanal	RI MS St	1286	1279	0.07 ± 0.01 d	0.16 ± 0.01 c	0.29 ± 0.01 a	0.2 ± 0.03 b
B4	Nonanal	RI MS St	1390	1384	0.00 ± 0.00 d	0.04 ± 0.00 b	0.06 ± 0.01 a	0.02 ± 0.00 c
B5	Decanal	RI MS St	1472	1433	0.5 ± 0.02 a	0.25 ± 0.01 b	0.25 ± 0.03 b	0.18 ± 0.02 c
B6	Benzaldehyde	RI MS St	1502	1508	0.01 ± 0.00	ND	ND	ND
B7	Undecanal	RI MS St	1622	1613	0.19 ± 0 a	ND	0.10 ± 0.07 b	0.11 ± 0.00 ab
C1	Linalool	RI MS St	1544	1550	0.38 ± 0.02 a	0.04 ± 0.00 c	0.22 ± 0.02 b	0.16 ± 0.00 b
C2	(*Z*)-2-Penten-1-ol	RI MS St	1304	1315	0.05 ± 0.00	ND	ND	ND
C3	Hexanol	RI MS St	1345	1351	67.76 ± 0.55 a	46.49 ± 0.45 d	56.98 ± 1.29 b	54.67 ± 0.12 c
C4	(*Z*)-3-Hexen-1-ol	RI MS St	1378	1378	20.45 ± 0.11 a	11.28 ± 0.34 c	15.84 ± 0.42 b	15.24 ± 0.13 b
C5	2,4-Hexadien-1-ol	RI MS	1523	1500	ND	ND	ND	0.01 ± 0.00
C6	1-Octen-3-ol	RI MS St	1430	1447	0.06 ± 0.00 a	0.01 ± 0.00 d	0.05 ± 0.00 b	0.03 ± 0.00 c
C7	Heptanol	RI MS St	1447	1451	0.11 ± 0.01 a	ND	ND	0.07 ± 0.00 b
C8	(*Z*)-2-Hepten-1-ol	MS	-	1475	ND	ND	0.06 ± 0.01	ND
C9	(*E*)-2-Hepten-1-ol	RI MS	1517	1517	0.02 ± 0.00	ND	ND	ND
C10	Octanol	RI MS St	1550	1558	0.15 ± 0.01 a	0.18 ± 0.02 a	0.16 ± 0.00 a	0.10 ± 0.00 b
C11	Verbenol	RI MS	1675	1680	0.09 ± 0.01 a	ND	ND	0.05 ± 0.00 b
C12	Terpinen-4-ol	RI MS St	1586	1591	0.30 ± 0.00 b	0.39 ± 0.00 a	0.39 ± 0.01 a	0.29 ± 0.01 b
C13	(*E*)-2-Octen-1-ol	MS	-	1606	0.18 ± 0.01 a	0.06 ± 0.00 b	0.18 ± 0.00 a	0.17 ± 0.02 a
C14	Nonanol	RI MS St	1640	1649	0.27 ± 0.01	0.55 ± 0.01	ND	ND
C15	*α*-Terpineol	RI MS St	1669	1684	1.31 ± 0.02 d	1.93 ± 0.03 a	1.77 ± 0.09 b	1.60 ± 0.03 c
D1	6-Methyl-5-Hepten-2-one	RI MS St	1323	1327	0.04 ± 0.02 d	0.06 ± 0.00 c	0.18 ± 0.01 a	0.13 ± 0.00 b
D2	2-Nonanone	RI MS St	1387	1367	ND	0.90 ± 0.07 b	0.94 ± 0.04 b	1.15 ± 0.03 a
E1	Hexyl acetate	RI MS St	1267	1308	ND	0.16 ± 0.00 a	0.16 ± 0.03 a	0.13 ± 0.01 a
E2	(*Z*)-2-Hexen-1-ol acetate	RI MS	-	1302	ND	ND	ND	0.02 ± 0.00
F1	3-Octenoic acid	MS	-	1401	0.04 ± 0.00 a	0.03 ± 0.00 c	0.04 ± 0.00 c	0.04 ± 0.00 b

One-way analysis of variance (ANOVA) was applied to compounds in four samples: Roman letters (a–d) mean significant differences among samples. RI: The retention indices of the identified compounds were consistent with those recorded in the NIST webbook; MS: Mass spectra of the identified compounds corresponded with those recorded in the NIST library; St: Confirmed by comparing the retention time of standard aroma compounds in GC-MS; ND: Not detected in samples; RIa: Retention index queried using the flavornet database (https://webbook.nist.gov/chemistry/) (accessed on 2 December 2023); RIb: Retention index obtained on DB-WAX column via GC-MS with a homologous series of n-alkanes (C_7_–C_40_).

## Data Availability

The original contributions presented in this study are included in the article. Further inquiries can be directed to the corresponding author.

## Abbreviations

The following abbreviations are used in this manuscript:

HCA	Hierarchical clustering analysis
GC-MS	Gas chromatography–mass spectrometry
HS-SPME	Headspace solid-phase microextraction
NIST	National Institute of Standards and Technology
OPLS-DA	Orthogonal partial least squares–discriminant analysis
PCA	Principal component analysis
OAV	Odor activity value
VIP	Variables important in the projection
